# Evaluation of Retinal Function and Pathology After Intravitreal Injection of Povidone-Iodine and Polyvinyl Alcohol-Iodine in Rabbits

**DOI:** 10.1167/tvst.9.5.5

**Published:** 2020-04-15

**Authors:** Hiroyuki Shimada, Kimiko Kato, Kazuumi Ishida, Takanori Yamaguchi, Kei Shinoda

**Affiliations:** 1 Department of Ophthalmology, Nihon University Hospital, Chiyoda-ku, Tokyo, Japan; 2 R&D Department, Nitten Pharmaceutical Co., Ltd., Nagoya, Aichi, Japan; 3 Department of Ophthalmology, Faculty of Medicine, Saitama Medical University, Iruma, Saitama, Japan

**Keywords:** electroretinography, retinal toxicity, pathological examination, polyvinyl alcohol-iodine, povidone-iodine

## Abstract

**Purpose:**

This study compared intraocular toxicity of intravitreally injected povidone-iodine (PI) and polyvinyl alcohol-iodine (PAI) in rabbits.

**Methods:**

In each rabbit, 0.1 mL of PI or PAI solution was injected intravitreally into one eye and saline was injected into the other. PI was tested at available iodine concentrations of 0.05%, 0.1%, 0.2%, and 0.5%, and PAI at 0.05%, 0.1%, and 0.2% (n = 6 each). Electroretinograms were recorded before injection and 1, 7, and 14 days after injection. Pathological examinations of eyeballs were performed on day 15.

**Results:**

Mean b-/a-wave ratios of the electroretinograms did not change in eyes injected with 0.05%, 0.1%, or 0.2% PI (PI-0.05, PI-0.1, and PI-0.2, respectively) or in eyes injected with 0.05% or 0.1% PAI (PAI-0.05 and PAI-0.1, respectively) compared to saline-injected eyes, but was transiently impaired on day 1 in PAI-0.2 eyes. Histopathologically, no retinal abnormalities were observed in PI-0.05, PAI-0.05, or PAI-0.1 eyes. One PI-0.1 eye first showed localized inflammatory cell infiltration in the inferior retinal region. Two PI-0.2 eyes and one PAI-0.2 eye had retinal degeneration and inflammatory cell infiltration. In the PI-0.5 group, extensive inflammatory cell infiltration was observed in six eyes and inferior retinal detachment in five eyes.

**Conclusions:**

PI and PAI have equivalent retinal toxicity profiles, and retinal toxicity first affects the inner retinal layer in the inferior region. The highest non-retinotoxic vitreous concentration is 0.0033% available iodine from intravitreal injection of PI or PAI containing 0.05% available iodine.

**Translational Relevance:**

Low concentrations of PI or PAI can be used to wash the ocular surface during surgery or intravitreal injection to prevent endophthalmitis.

## Introduction

The conjunctiva contains resident bacteria that protect the eye from invasion by pathogens. Many resident bacteria are non-pathogenic in the conjunctiva but become pathogenic when transferred from the conjunctiva into the eye. In eyes with postoperative endophthalmitis, typing and genetic testing of the causative microorganisms have confirmed that the normal flora inhabiting the eyelid and conjunctiva of the operated eye are altered.^1^^–^^3^ Therefore, for cataract surgery, vitrectomy and intravitreal injection, which are procedures that communicate the conjunctiva with the inside of the eye, the risk of endophthalmitis caused by introduction of resident bacteria into the eye cannot be eliminated.^4^^,^^5^

As a prophylactic measure against endophthalmitis, preoperative washing with povidone-iodine (PI) has the highest level of evidence compared with preoperative instillation of antimicrobial agent and addition of antimicrobial agent into the perfusion solution.^6^^,^^7^ Furthermore, PI did not promote bacterial resistance or alter conjunctiva flora in one study.^8^ Several articles have also shown that washing the ocular surface with a diluted solution of PI during surgery achieves temporary sterilization of the ocular surface and that preoperative washing with the solution prevents the invasion of resident bacteria into the eye.^9^^–^^11^

Both PI and polyvinyl alcohol-iodine (PAI) are intermediate-level, iodine-based disinfectants that kill microbes (other than spores), including drug-resistant bacteria,^12^ viruses,^13^ fungi,^14^ and *Acanthamoeba*,^15^ and they are also active against biofilm.^16^ Diluted PAI is as effective as PI in killing bacteria, and cleaning the ocular surface with PAI every 20 to 30 seconds during cataract surgery has been reported to result in an extremely low rate of bacteria detection in the anterior chamber at the end of surgery, without damage to corneal endothelial cells.^17^

In a solution of PI or PAI, free iodine is released which acts directly on the membrane proteins of bacteria and virus to exhibit a microbicidal effect.^11^^,^^18^^,^^19^ However, iodine also has been reported to cause damage to corneal epithelial cells,^20^^–^^23^ corneal endothelial cells,^23^^–^^26^ and retina.^27^^–^^29^ Because it also acts directly on the membrane proteins of normal cells, identifying the safe and at the same time effective concentration range becomes important.^11^^,^^18^^,^^30^^,^^31^

Until recently, iodine has been used only for disinfection of the ocular surface prior to surgery. Recently, the method of washing the surface of the eye with PI or PAI every 20 to 30 seconds during cataract or vitreous surgery has become widely used mainly in Japan.^9^^–^^11^^,^^17^^,^^19^ However, irrigation of the ocular surface with dilute PI or PAI during surgery has a risk of accidentally introducing the solution into the eye. For this reason, it is important to examine the retinal toxicity of iodine in detail. There are several reports on the effects of PI on the retina,^27^^–^^29^ but no reports for PAI. In this study, we compared the effects of both PI, which is widely used throughout the world, and PAI, which is commonly used in Japan, on rabbit eyes using electroretinography (ERG) and pathological examination.

## Materials and Methods

### Animals

Forty-two male Japanese white rabbits (Japan SLC, Shizuoka, Japan) weighing approximately 2 to 3 kg were used. They were divided into seven groups (n = 6 each). All experimental procedures were performed in accordance with the ARVO Statement for the Use of Animals in Ophthalmic and Vision Research and the experimental protocol. All protocols were approved by the Laboratory Animal Committee of Nitten Pharmaceutical Co., Ltd.

### Intravitreal Injection

The rabbits were anesthetized with intramuscular injection of 25 mg/kg ketamine and 2 mg/kg xylazine. Iodine preparation or physiological saline was injected into the vitreous using a 27-gauge needle inserted approximately 2 mm posterior to the limbus in the superior temporal quadrant. The PI product used was Isodine solution 10% (Mundipharma International Ltd., Cambridge, UK), and the PAI product was PA Iodo Ophthalmic and Eye Washing Solution (Nitten Pharmaceutical Co., Ltd., Nagoya, Japan). The PI product contains 1% available iodine, and the PAI product contains 0.2% available iodine. The two products were tested at equivalent available iodine concentrations. The PAI solutions tested contained 0.2% (neat solution; PAI-0.2 eyes), 0.1% (1:2 dilution; PAI-0.1 eyes), and 0.05% available iodine (1:4 dilution; PAI-0.05 eyes). For PI, an additional higher iodine concentration was tested. Thus, the PI solutions tested contained 0.5% (1:2 dilution; PI-0.5 eyes), 0.2% (1:5 dilution; PI-0.2 eyes), 0.1% (1:10 dilution; PI-0.1 eyes), and 0.05% available iodine (1:20 dilution; PI-0.05 eyes). PI and PAI were diluted with physiological saline. In each rabbit, 0.1 mL of saline was injected intravitreally into one eye (right eye), and 0.1 mL of test solution was injected into the contralateral eye (left eye).

### Ophthalmological Examination

Using a direct ophthalmoscope (Welch Allyn, Skaneateles Falls, NY, USA), the anterior and posterior segments of the rabbits’ eyes were examined under mydriasis with 0.5% tropicamide and 0.5% phenylephrine. All animals were examined using a hand-held slit lamp (Welch Allyn) and ophthalmoscope (Welch Allyn) before intravitreal injection, immediately after injection, and 1, 7, 14 (before dark adaptation), and 15 days after injection.

### ERG Recordings

ERGs were recorded according to the protocols of the International Society for Clinical Electrophysiology of Vision.^32^ After mydriasis with eye drops containing 0.5% tropicamide and 0.5% phenylephrine and dark adaptation for 60 minutes, the animal was anesthetized with intramuscular injection of 25 mg/kg ketamine and 2 mg/kg xylazine. After 60 minutes of dark adaptation, the cornea was anesthetized with eye drops containing 0.4% oxybuprocaine hydrochloride. The rabbit was restrained, and electrode placement was performed under dim red light. Then, ERGs were recorded using a contact lens electrode with built-in light-emitting diodes (HW-S6; Mayo, Inc., Nagoya, Japan) and an evoked response recording device (Model PuRec; Mayo, Inc.). Rod responses in dark fields were recorded at a light intensity of 0.01 cd·s·m^−2^. Mixed responses were recorded at light intensities of 3 and 10 cd·s·m^−2^. Oscillatory potentials (OPs) were recorded at a light intensity of 3 cd·s·m^−2^. After 10 minutes of bright adaptation, bright-field cone responses were recorded at a light intensity of 3 cd·s·m^−2^ with background light of 30 cd·m^−2^. Then, 30-Hz flicker responses were recorded at a light intensity of 3 cd·s·m^−2^ with background light of 30 cd·m^−2^. ERGs were recorded using a digital band-pass filter in the range of 0.3 to 300 Hz for both dark- and bright-field ERGs, except for OPs, which were extracted with a 75- to 300-Hz digital band-pass filter. ERGs were recorded before injection and 1, 7, and 14 days after intravitreal injection.

### Histopathological Examination

At day 15 after intravitreal injection, animals were given intravenous pentobarbital sodium (Somnopentyl; Kyoritsu Seiyaku Corp., Tokyo, Japan) and euthanized by exsanguination. The enucleated eyeballs were incised vertically at the temporal side and then immersed in 1% buffered formaldehyde with 2.5% glutaraldehyde. The next day, they were transferred to 10% buffered formalin. Tissues were paraffin-embedded, sectioned at 4 µm, and stained with hematoxylin and eosin (HE). Histological examinations of the eyeball including the retina, optic nerve, cornea, anterior chamber angle, and lens were performed using a light microscope.

### Statistical Analyses

The amplitudes and the implicit times of ERG were calculated as the ratio of the left to right eye of the same animal (iodine-injected/saline-injected). Statistical analyses were performed using GraphPad Prism 7 (Graph Pad Software, San Diego, CA, USA). Statistical comparisons of the amplitude ratio and implicit time ratio among four time periods (before injection and 1, 7, and 14 days after injection) were performed by Bonferroni multiple comparisons. *P* < 0.05 was considered statistically significant (multiplicity-adjusted *P* values). Comparison of the b-/a-wave ratio between eyes was performed by paired *t*-test, and *P* < 0.05 was considered statistically significant.

## Results

Physiological saline and iodine preparations (PI-0.05, PI-0.1, PI-0.2, PI-0.5, PAI-0.05, PAI-0.1, and PAI-0.2) were injected intravitreally into rabbits to evaluate the effects of the solutions on the retina. ERG recordings were performed before injection and 1, 7, and 14 days after injection. Histological examination was performed at day 15, after finishing the 14-day ERG follow-up.

### Ophthalmological Examination

**Figure 1.**
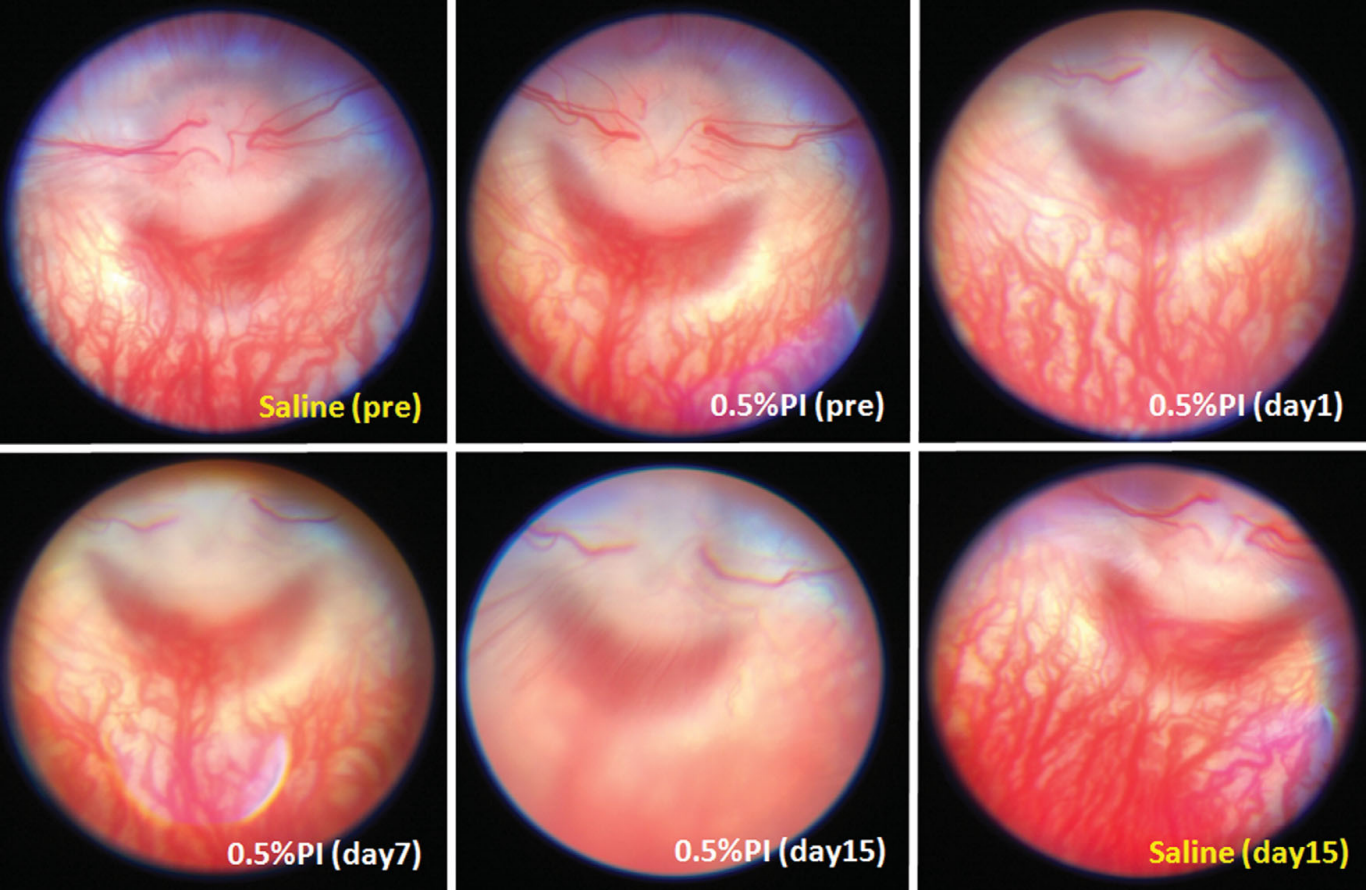
Serial fundus images of a rabbit before and after intravitreal injection of povidone-iodine containing 0.5% available iodine (PI-0.5). The contralateral control eye was injected with saline. No abnormality was observed 1 day after the 0.5% PI injection, but optic nerve atrophy and retinal detachment of the lower optic disc were observed 15 days after injection.

The fundus, cornea, and lens of each eye were examined before injection, immediately after intravitreal injection, and 1, 7, 14 and 15 days after injection. Regardless of the injected concentration, PI or PAI was clearly visible following injection as a brown cloud concentrated in the posterior vitreous, which spread over the entire posterior pole within several minutes. Although the visual evoked potential was not recorded, ophthalmoscopic images were recorded for all injected eyes on days 0, 1, 7, and 14 and just before enucleation on day 15. No abnormalities were observed after injection in saline-injected eyes; in PI-0.05, PI-0.1, and PI-0.2 eyes; or in PAI-0.05, PAI-0.1, and PAI-0.2 eyes until day 15. In PI-0.5 eyes, no abnormalities were observed immediately after injection, but optic atrophy and retinal detachment in the region inferior to the optic disc were observed in five of six eyes at 15 days after injection (Fig. 1).

### Electrophysiological Tests

**Figure 2.**
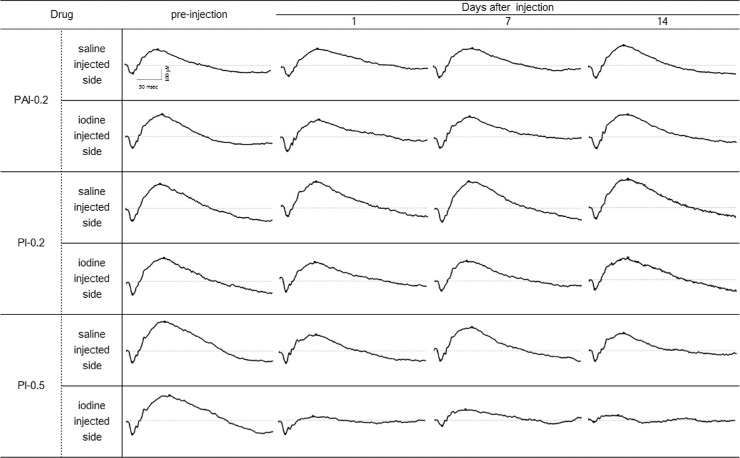
ERG waves after intravitreal injection of iodine preparation or saline (control eyes) in rabbits. The ERGs recorded before injection and 1, 7, and 14 days after injection for each agent belonged to the same rabbit. Injection of PAI and PI containing 0.2% available iodine did not alter the ERG waves compared with before injection or with saline injection. For PI containing 0.5% available iodine (PI-0.5 eyes), at 1 day after injection the a-wave amplitudes were not changed, but the b-wave amplitudes were reduced by an average of 30% (*P* < 0.05) compared with saline injection.

ERGs were recorded before injection and 1, 7, and 14 days after intravitreal injection. Various components of ERG waves were analyzed. Especially, amplitudes of a-waves derived from the outer retinal layers, amplitudes of b-waves derived from the middle retinal layers, and their ratios (b-/a-wave ratios) were compared among groups. Representative examples of ERG waves are shown in Figure 2. The ERG waves were not altered in the PI-0.2, PI-0.1, PI-0.05, PAI-0.2, PAI-0.1, and PAI-0.05 eyes compared to before injection. In PI-0.5 eyes, the only group injected with a high available iodine concentration of 0.5%, the amplitudes of a-waves were not reduced compared to before injection at 1 day after injection but decreased by 41% (*P* < 0.01) and 62% (*P* < 0.01) at 7 and 14 days, respectively (Supplementary Table S1; 3 cd). On the other hand, the amplitudes of b-waves were reduced by an average of 30% (*P* < 0.05) compared to before injection at 1 day after injection and further to 50% (*P* < 0.01) and 64% (*P* < 0.01) at 7 and 14 days, respectively. The amplitudes of OP waves were not reduced compared to before injection at 1 and 7 days after injection but decreased by 45% (*P* < 0.01) at 14 days.

**Figure 3.**
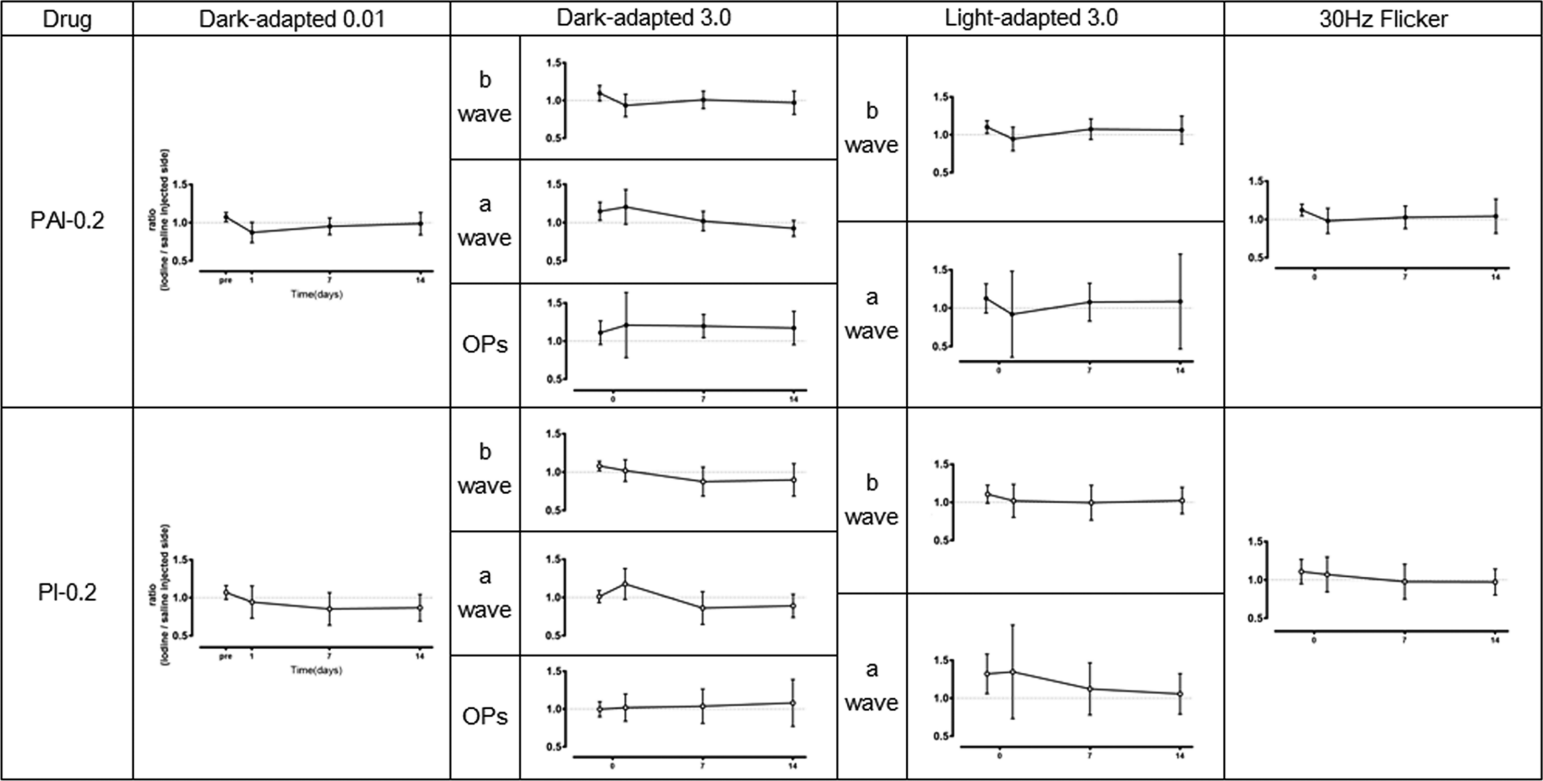
Time courses of amplitudes of waves on ERGs induced by light or dark adaptation. There were no great differences between PAI or PI containing 0.2% available iodine and saline for all components of the ERGs. There were no significant differences in the changes over time after injection compared with before injection (Bonferroni multiple comparisons). Data are presented as mean (open or closed circle) and standard deviation (vertical bar).

Figure 3 shows the ERGs results recorded over time at different measurement conditions in PI-0.2 and PAI-0.2 eyes. In order to analyze the effects of iodine preparations on the components of ERGs, ERG parameters were presented as the ratios of left to right eye of the same animal (iodine-injected/saline-injected). There were no great differences between PI-0.2 or PAI-0.2 eyes and saline-injected eyes in all components of the ERGs, and there were no significant differences in the changes over time after injection compared to before injection.

The mean b-/a-wave ratio of ERGs in PI-0.05, PI-0.1, PI-0.2, PAI-0.05, and PAI-0.1 eyes did not change compared to saline-injected eyes (Supplementary Table S2). However, PAI-0.2 eyes showed mild but significant transient impairment on day 1 (3 cd and 10 cd; *P* < 0.05). On the other hand, although PI-0.2 eyes showed mild transient reductions, the difference did not reach statistical significance. In PI-0.5 eyes, the mean b-/a-wave ratios in the iodine-injected eyes at 1 day after injection were 1.87 in 3 cd and 1.94 in 10 cd and were markedly and significantly smaller than in the saline-injected eyes (*P* < 0.01).

The implicit time showed no significant difference between iodine-injected eyes and saline-injected eyes in all groups except the PI-0.5 group. In the PI-0.5 group, the implicit times of b-waves, expressed as the ratio of left eye to right eye of the same animal (iodine-injected/saline-injected), in light-adapted 3.0 ERGs and flicker ERGs were significantly larger at various time points after injection compared to before injection (Supplementary Table S3).

### Pathological Examination

**Figure 4.**
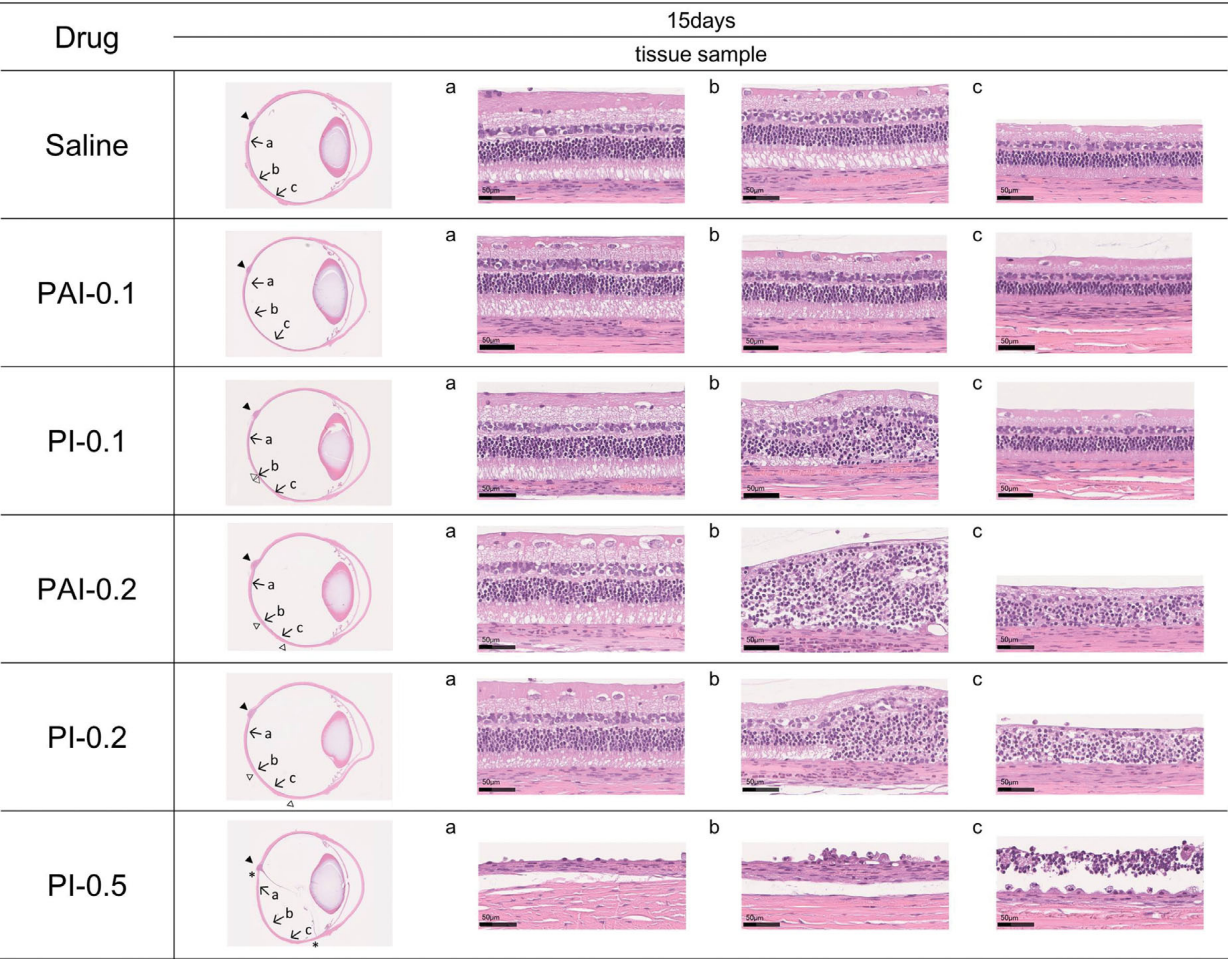
HE-stained sections of ocular tissue 15 days after intravitreal injection of PAI containing 0.1% and 0.2% available iodine (PAI-0.1 and PAI-0.2, respectively) and PI containing 0.1%, 0.2%, and 0.5% available iodine (PI-0.1, PI-0.2, and PI-0.5, respectively) in rabbits. Sections of retina on the right correspond to the locations of the arrows marked in the vertical section of the eyeball: (a) central retina, (b) region inferior to central retina, and (c) peripheral retina. The closed arrowhead (▲) shows the position of the optic disc. In the PI-0.5 group, the area between two asterisks (*) indicates the region of retinal detachment. The area between two open arrowheads (△) indicates the region of inflammatory cell infiltration in the retina.

Figure 4 shows the HE-stained vertical sections of eyeballs and magnified micrographs of retinal sections at 15 days after intravitreal injection. In saline-injected eyes, no abnormalities were observed in the retina. In the PAI-0.05, PI-0.05, and PAI-0.1 groups, no eyes showed abnormalities in retina. In the PI-0.1 group, one of six eyes showed localized inflammatory cell infiltration in the region inferior to the central retina (Fig. 4b; open arrowheads). However, inflammatory cell infiltration was not observed in peripheral retina (Fig. 4c).

In the PI-0.2 and PAI-0.2 groups, there were no abnormalities in the central retina (Fig. 4a); however, two of six eyes in the PI-0.2 group and one of six eyes in the PAI-0.2 group showed inflammatory cell infiltration in the retina and retinal degeneration (Figs. 4b, 4c) in the region inferior to the central retina (Fig. 4; area between the two open arrowheads). In the region inferior to the central retina, the boundary between the region without inflammatory cell infiltration and the region with inflammatory cell infiltration was clear, and inflammatory cell infiltration was observed extending to the peripheral retina.

In the PI-0.5 group, extensive inflammatory cell infiltration was observed in all six eyes, and retinal detachment was observed in the region inferior to the optic disc in five of six eyes (Fig. 4; area between two asterisks). In addition, inflammatory cell infiltration on the vitreous side of the abnormal region was observed in four of six eyes.

## Discussion

The effects of intravitreal PAI injection on rabbit retina were compared with PI injection by ERG and pathology. We found that PI and PAI had equivalent retinal toxicity profiles, and retinal toxicity first affected the inner retinal layer in inferior region. We also demonstrated that the highest non-retinotoxic vitreous concentration was 0.0033% available iodine, when 0.1 mL of 0.05% PI or PAI was injected intravitreally.

PI was first developed in 1956^33^ and is widely used not only in Japan but also worldwide. PI exhibits wide-spectrum microbicidal actions, is low cost, does not induce drug resistance, and has rapid microbicidal action.^8^^,^^11^^,^^18^ In addition, the safe concentrations and toxicity for intraocular tissues have been studied in detail.^11^^,^^18^ On the other hand, PAI was developed in 1959^34^ and is widely used mainly in Japan.^35^^,^^36^ PAI contains 2 mg of iodine per mL (0.2% available iodine), and the addition of the surfactant polyvinyl alcohol (80 mg/mL) reduces irritation to the eye.

Previous studies have used ERG and retinal tissue pathology to evaluate retinal damage as an indicator of intraocular toxicity. Several studies have examined the effects of intravitreal PI on retinal function and tissue in rabbits. Trost et al.^28^ reported no effects on ERGs or retinal tissue following intravitreal injection 0.1 mL of PI with available iodine concentrations of 0.005%, 0.01%, 0.02%, and 0.04%. Kim et al.^29^ showed that single intravitreal injection of 0.1 mL of PI with available iodine concentrations of 0.01 and 0.03% did not adversely affect ERGs and histologic examination. Whitacre et al.^27^ injected 0.1 mL of PI with available iodine concentrations of 0.005%, 0.05%, and 0.5% intravitreally into rabbit eyes and found reduced ERG amplitudes and retinal tissue damage in 1 of 10 eyes at 0.05%. In this one eye, mild suppression (22%) of a- and b-waves of the ERG was seen one week after injection. Pathological examination of this eye revealed focal retinal edema and necrosis involving the visual streak and inferior retina. All four eyes injected with 0.5% developed temporary hypotony, iridocyclitis, and full thickness retinal necrosis. An immediate, profound (40%–80%) reduction of a- and b-waves was seen in all eyes. One day after injection, there was edema in the nerve fibers, ganglion cells, and inner plexiform layers of the retina. At 7 and 28 days, full-thickness necrosis of the sensory retina was observed.

**Figure 5.**
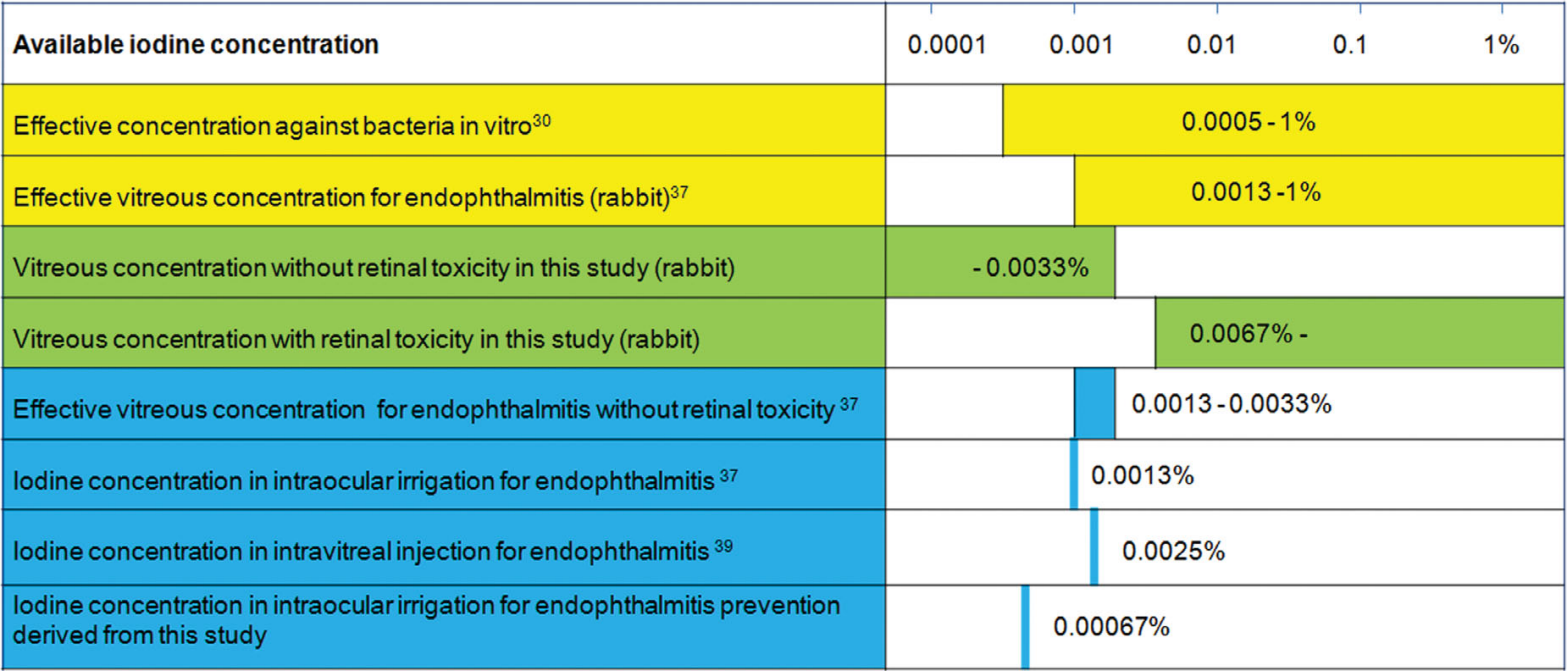
Summary of findings in the present and previous studies. Yellow bars indicate the results of previous efficacy studies, and green bars show the results of this research. Blue bars indicate the results derived from previous and current studies.

In the present study, ERG and pathological findings indicate that the highest vitreous concentration without retinal toxicity was due to intravitreal injection of 0.1 mL of 0.05% PI or 0.1% PAI. This amount is noteworthy, because when injected into the rabbit vitreous that has a volume of 1.5 mL, 0.1 mL of 0.05% available iodine concentration will be diluted to 0.0033% (Fig. 5). Brozou et al.^37^ reported that intravitreal injection of PI with 0.01% available iodine (0.00067% in rabbit vitreous) did not inhibit bacterial endophthalmitis, whereas PI with 0.02% available iodine (0.0013% in rabbit vitreous) was likely to inhibit bacterial endophthalmitis. From their findings and the results of this study, the effective vitreous concentration range for endophthalmitis without retinal toxicity can be calculated as 0.0013% to 0.0033%. This supports the clinical report of Nakashizuka et al.,^38^^,^^39^ who treated endophthalmitis with intravitreal injections of 0.1 mL of PI with 0.125% available iodine (0.0025% in 5 mL of human vitreous) followed by vitrectomy using 0.0025% PI in BSS PLUS (Alcon, Ft. Worth, TX, USA). For the treatment of endophthalmitis, intravitreal injection with 0.125% PI is a non-retinotoxic concentration; however, the PI added to the intraocular cleansing solution makes contact with intraocular tissues already damaged by endophthalmitis. Therefore, the lowest concentration of 0.0013% PI should be selected.^40^

If iodine can be added to the infusion fluid used in cataract and vitreous surgeries, it will be useful in preventing endophthalmitis. In this study, the initial retinal toxicity was observed with PI or PAI containing 0.1% available iodine (0.0067% in rabbit vitreous). Therefore, a 1/10 concentration, or 0.01% (0.00067% in rabbit vitreous), may be suitable for prevention of endophthalmitis.^41^^,^^42^

In previous studies, washing the ocular surface with a 0.025% available iodine concentration of PI (0.25% iodine concentration) during surgery has prevented the normal flora on the ocular surface from entering the eye.^9^^,^^10^ In the clinical setting, when 0.1 mL of PI or PAI containing 0.025% available iodine is introduced into human vitreous (0.0005% in 5 mL of human vitreous), the concentration is not considered to be retinotoxic.

In this study, no retinal abnormalities were observed in PAI-0.05, PI-0.05, and PAI-0.1 eyes. Among six PI-0.1 eyes, one eye showed localized inflammatory cell infiltration in the region inferior to the central retina. These results suggest that early retinal damage due to iodine occurs in the region inferior to the central retina. In PAI-0.2 and PI-0.2 eyes, an iodine concentration that has not been researched previously, inflammatory cell infiltration in the retina and destruction of retinal layer structure were observed in the region inferior to the central retina extending to the peripheral retina. In PI-0.5 eyes, extensive inflammatory cell infiltration and retinal detachment were observed in the region inferior to the optic disc.

Ocular tissue specimens are usually studied histopathologically by preparing horizontal sections of the eyeball. In this study, however, vertical sections were made to view the regions around of the central retina. The injected PAI or PI was clearly visible following injection as a brown cloud concentrated in the posterior vitreous, which spread over the entire posterior pole within several minutes. Kim et al.^29^ reported that the half-life of PI in the vitreous of rabbit eyes was approximately 3 hours; however, vitreous body of a healthy rabbit is highly viscous, and the rabbit always has the 12 o'clock position of the eyeball positioned upward. Therefore, the presence of relatively high concentration of iodine near the inferior region of the eye could have led to the development of local damage. In order to observe the retinal damage caused by intravitreal injection of the drug, it is important to observe the eyeball by vertical section centering on the optic disc.

Next, we analyzed the ERG findings in detail. The mean b-/a-wave ratio ERGs did not change in PI-0.05, PI-0.1, PI-0.2, PAI-0.05, or PAI-0.1 eyes compared to saline-injected eyes. Although PAI-0.2 eyes showed transient mild but significant reduction on day 1, PI-0.2 eyes showed transient mild reduction that was not statistically significant. These results showed that 0.2% injection of PI or PAI caused transient mild electrophysiological dysfunction to the middle retinal layers. In contrast, the b-/a-wave ratio in PI-0.5 eyes was markedly and significantly reduced compared to saline-injected eyes at 1 day after injection, due to significant decreases in b-wave amplitudes but no change in a-waves. Thereafter, the b-/a-wave ratio increased with time to levels similar to those in saline-injected eyes. This was not due to improvement of function in the middle layers but instead was caused by significant decrease of function in the outer layers together with continued decline in the middle layers. Iodine injected into the vitreous is thought to affect the inner layer of the retina first and subsequently the outer layer of the retina. Because we performed pathological examinations on the 15th day after iodine injection, we assumed that the results reflected the end stage of the effects of iodine-induced retinal toxicity. Thus, we were not able to follow the changes over time. Moreover, there are no reports of iodine-induced retinal pathology observed over time. Because optic nerve head atrophy was observed in PI-0.5 eyes at day 15, further histopathological study is needed to examine the initial lesion and subsequent transition of retinal toxicity over time after intravitreal injection of iodine. In addition the visual evoked potential would be useful to detect initial alteration of optic nerve function. Using techniques such as optical coherence tomography, it may be possible to observe the temporal changes in the layer structure of the entire retina.

## Conclusions

The present study demonstrates that PI and PAI have equivalent retinal toxicity profiles following intravitreal injection. Retinal toxicity first affects the inner retinal layer in the inferior region. The highest non-retinotoxic vitreous concentration is 0.0033% available iodine, by intravitreal injection of 0.1 mL of PI or PAI containing 0.05% available iodine.

## Supplementary Material

Supplement 1

Supplement 2

Supplement 3
