# Supplementary material for: Evaluation of Retinal Function and Pathology After Intravitreal Injection of Povidone-Iodine and Polyvinyl Alcohol-Iodine in Rabbits
Source: Transl Vis Sci Technol. 2020 Apr 15;9(5):5. doi: 10.1167/tvst.9.5.5 (PMC7401888; doi:10.1167/tvst.9.5.5)
Supplement: Supplement 1 [file tvst-9-5-5_s001.pdf]

Table 1 Ratios of wave amplitude in iodine-injected eye to saline-injected control eye for various ERG components induced by various stimuli before intravitreal injection and 1, 7 and 14 days after injection.

Group	Day	Dark-adapted						Light-adapted		
		0.01 cd·s·m^{-2}	3.0 cd·s·m^{-2}			10.0 cd·s·m^{-2}		3.0 cd·s·m^{-2}		30Hz Flicker
			a-wave	b-wave	OPs	a-wave	b-wave	a-wave	b-wave	
PAI-0.05	pre	0.97±0.10	1.00±0.08	0.98±0.06	0.96±0.10	1.00±0.09	0.97±0.07	0.93±0.23	0.99±0.10	1.00±0.09
	1	1.04±0.24	1.07±0.22	1.03±0.21	1.10±0.26	1.16±0.24	1.11±0.24	1.79±1.01	1.15±0.27	1.10±0.26
	7	0.98±0.12	1.10±0.36	1.00±0.15	1.07±0.15	1.00±0.13	1.13±0.33	1.38±0.71	1.12±0.19	1.04±0.16
	14	0.94±0.11	0.93±0.12	0.97±0.06	0.89±0.11	0.99±0.12	0.96±0.11	1.09±0.23	0.94±0.06	0.97±0.09
PAI-0.1	pre	1.00±0.05	1.01±0.09	0.98±0.08	1.02±0.07	1.05±0.04	1.02±0.06	1.09±0.43	1.04±0.08	1.06±0.10
	1	0.97±0.15	0.95±0.10	1.01±0.13	0.91±0.20	0.99±0.04	0.99±0.16	0.85±0.18	0.94±0.07	0.99±0.12
	7	1.05±0.12	1.06±0.14	1.06±0.11	1.10±0.18	1.10±0.09	1.07±0.10*	1.15±0.65	1.12±0.14	1.15±0.32
	14	0.99±0.18	0.98±0.12	1.02±0.16	1.08±0.20	1.04±0.12	1.03±0.13	1.16±0.31	1.01±0.13	1.05±0.17
PAI-0.2	pre	1.07±0.06	1.15±0.12	1.10±0.10	1.11±0.15	1.12±0.12	1.06±0.08	1.13±0.19	1.10±0.08	1.12±0.08
	1	0.87±0.13	1.20±0.23	0.93±0.15	1.21±0.43	1.25±0.42	0.94±0.16	0.92±0.56	0.94±0.16	0.98±0.16
	7	0.95±0.11	1.02±0.13	1.01±0.12	1.20±0.15	1.02±0.12	1.04±0.12	1.08±0.25	1.08±0.14	1.03±0.15
	14	0.98±0.15	0.93±0.10	0.97±0.16	1.17±0.22	1.01±0.15	0.93±0.13	1.09±0.62	1.06±0.19	1.04±0.22
PI-0.05	pre	0.97±0.08	0.98±0.13	0.98±0.09	1.07±0.15	0.99±0.13	0.98±0.10	1.33±0.41	1.05±0.16	1.03±0.09
	1	0.98±0.09	1.04±0.17	1.03±0.08	0.96±0.12	1.09±0.17	1.03±0.05	1.07±0.36	1.06±0.11	1.16±0.12
	7	0.98±0.09	1.00±0.11	1.01±0.18	1.01±0.13	0.97±0.10	1.01±0.08	1.01±0.40	1.07±0.09	1.06±0.09
	14	1.01±0.07	1.01±0.16	1.08±0.07	1.03±0.17	1.05±0.20	1.06±0.10	1.16±0.48	1.06±0.14	1.07±0.22
PI-0.1	pre	1.03±0.06	1.04±0.11	1.07±0.08	1.07±0.15	1.06±0.12	1.08±0.06	1.31±0.28	1.10±0.12	1.07±0.15
	1	1.03±0.11	1.10±0.10	1.01±0.10	0.93±0.18	1.02±0.11	0.97±0.06	0.92±0.22	1.01±0.12	1.00±0.12
	7	0.99±0.20	0.99±0.16	1.01±0.18	1.01±0.24	1.04±0.15	1.04±0.17	1.45±0.39	1.06±0.19	1.06±0.15
	14	1.00±0.09	0.98±0.07	0.99±0.13	1.14±0.13	1.07±0.19	1.04±0.14	0.82±0.25	1.06±0.13	1.03±0.06
PI-0.2	pre	1.07±0.09	1.01±0.08	1.08±0.06	1.00±0.10	1.12±0.07	1.09±0.10	1.32±0.26	1.11±0.12	1.11±0.16
	1	0.94±0.21	1.18±0.20	1.02±0.14	1.02±0.18	1.25±0.18	1.08±0.23	1.35±0.62	1.02±0.22	1.07±0.23
	7	0.85±0.21	0.86±0.22	0.87±0.19	1.04±0.23	0.88±0.19	0.90±0.20	1.12±0.34	0.99±0.23	0.97±0.23
	14	0.87±0.18	0.89±0.15	0.90±0.21	1.08±0.31	0.89±0.14	0.85±0.16	1.06±0.27	1.02±0.18	0.97±0.17
PI-0.5	pre	1.03±0.09	1.07±0.05	1.05±0.06	1.06±0.13	1.03±0.06	1.01±0.07	1.11±0.30	1.04±0.06	1.02±0.04
	1	0.68±0.22**	1.24±0.29	0.74±0.22*	0.88±0.25	1.19±0.34	0.83±0.24	0.74±0.20	0.74±0.24	0.85±0.29
	7	0.53±0.07**	0.63±0.19**	0.53±0.06**	0.75±0.12	0.60±0.08*	0.56±0.12**	0.61±0.19*	0.63±0.09**	0.62±0.07*
	14	0.34±0.18**	0.41±0.21**	0.38±0.19**	0.58±0.29**	0.51±0.25**	0.41±0.17**	0.58±0.39*	0.45±0.28**	0.37±0.30**

ERGs were recorded according to the stimulus conditions of International Society for Clinical Electrophysiology of Vision guideline. Each component of the ERGs was calculated as the ratio of the left to right eye of the same animal (iodine-injected side / saline-injected side). Data are expressed as mean ± standard deviation of 6 animals. *$P < 0.05$ **$P < 0.01$, significantly different from each pre-injection by Bonferroni multiple comparisons. PAI = polyvinyl alcohol-iodine; PI = povidone iodine; -0.05, -0.1, -0.2, -0.5 = 0.05%, 0.1%, 0.2%, 0.5% of available iodine, respectively; pre = pre-injection; OPs = oscillatory potentials
