# Supplementary material for: Evaluation of Retinal Function and Pathology After Intravitreal Injection of Povidone-Iodine and Polyvinyl Alcohol-Iodine in Rabbits
Source: Transl Vis Sci Technol. 2020 Apr 15;9(5):5. doi: 10.1167/tvst.9.5.5 (PMC7401888; doi:10.1167/tvst.9.5.5)
Supplement: Supplement 2 [file tvst-9-5-5_s002.pdf]

Table 2 The b/a ratios of dark-adapted 3.0 ERG in iodine-injected eyes and saline-injected control eyes before intravitreal injection and 1, 7 and 14 days after injection.

Group	Day	Dark-adapted 3.0 cd·s·m^{-2}		Dark-adapted 10.0 cd·s·m^{-2}	
		b/a ratio		b/a ratio	
		Saline-injected eye	Iodine-injected eye	Saline-injected eye	Iodine-injected eye
PAI-0.05	pre	3.33±0.20	3.29±0.23	3.15±0.15	3.06±0.12*
	1	3.24±0.37	3.13±0.46	3.17±0.42	3.06±0.51
	7	3.50±0.55	3.27±0.42	3.16±0.30	3.54±0.63
	14	3.53±0.22	3.72±0.51	3.28±0.48	3.17±0.46
PAI-0.1	pre	3.27±0.30	3.20±0.52	3.03±0.40	2.96±0.52
	1	2.99±0.53	3.14±0.45	2.92±0.70	2.89±0.61
	7	3.35±0.47	3.36±0.56	2.99±0.16	2.94±0.27
	14	3.12±0.29	3.25±0.33	3.26±0.52	3.22±0.37
PAI-0.2	pre	3.27±0.54	3.11±0.26	3.04±0.22	2.91±0.31
	1	3.26±0.30	2.58±0.50*	3.14±0.34	2.43±0.36*
	7	3.04±0.37	3.00±0.32	2.91±0.38	2.96±0.38
	14	3.05±0.35	3.17±0.31	3.07±0.34	2.81±0.27
PI-0.05	pre	3.28±0.54	3.29±0.47	3.06±0.46	3.04±0.46
	1	3.26±0.29	3.31±0.64	3.09±0.46	2.98±0.63
	7	3.41±0.19	3.47±0.51	3.17±0.47	3.31±0.43
	14	3.43±0.49	3.69±0.58	3.24±0.41	3.29±0.17
PI-0.1	pre	3.15±0.23	3.27±0.33	2.95±0.33	3.01±0.34
	1	3.37±0.24	3.11±0.41	3.06±0.31	2.93±0.33
	7	3.03±0.25	3.11±0.42	3.19±0.26	3.17±0.37
	14	3.31±0.31	3.38±0.55	3.19±0.28	3.11±0.37
PI-0.2	pre	3.05±0.52	3.24±0.48	2.92±0.28	2.84±0.29
	1	2.96±0.45	2.59±0.55	2.82±0.45	2.44±0.55
	7	3.15±0.24	3.23±0.39	3.02±0.37	3.10±0.31
	14	3.25±0.27	3.28±0.54	3.01±0.19	2.89±0.23
PI-0.5	pre	3.38±0.27	3.31±0.32	3.21±0.18	3.18±0.31
	1	3.14±0.53	1.87±0.41**	2.73±0.41	1.94±0.46**
	7	3.40±0.36	2.98±0.47	3.04±0.47	2.82±0.43
	14	3.49±0.43	3.27±1.01	3.16±0.24	2.69±0.59

Data are expressed as mean ± standard deviation of 6 animals.

*$P < 0.05$, **$P < 0.01$; significantly different from each saline-injected eye by paired t-test.

PAI = polyvinyl alcohol-iodine, PI = povidone iodine, -0.05, -0.1, -0.2, -0.5 = 0.05%, 0.1%, 0.2%, 0.5% of available iodine concentrations, respectively; pre = pre-injection
