# Supplementary material for: Evaluation of Retinal Function and Pathology After Intravitreal Injection of Povidone-Iodine and Polyvinyl Alcohol-Iodine in Rabbits
Source: Transl Vis Sci Technol. 2020 Apr 15;9(5):5. doi: 10.1167/tvst.9.5.5 (PMC7401888; doi:10.1167/tvst.9.5.5)
Supplement: Supplement 3 [file tvst-9-5-5_s003.pdf]

Table 3 Implicit time of ERGs

Group	Day	Dark-adapted					Light-adapted		
		0.01 cd･s･m^{-2}	3.0 cd･s･m^{-2}		10.0 cd･s･m^{-2}		3.0 cd･s･m^{-2}		30Hz Flicker
			a-wave	b-wave	a-wave	b-wave	a-wave	b-wave	
PI-0.5	pre	1.01±0.03	0.99±0.07	1.01±0.04	0.97±0.08	0.98±0.05	0.95±0.08	0.99±0.01	0.99±0.01
	1	0.96±0.04	1.07±0.13	1.00±0.10	1.03±0.06	1.01±0.09	1.02±0.07	1.09±0.02**	1.06±0.03*
	7	0.96±0.05	1.03±0.10	0.86±0.15	0.96±0.03	0.87±0.12	1.04±0.14	1.07±0.02**	1.03±0.03
	14	not available #	0.91±0.09	not available #	0.95±0.05	not available #	not available #	1.08±0.06**	1.06±0.06*

Implicit time of ERGs were calculated as the ratio of the left to right eye of the same animal (iodine-injected / saline-injected)

Data are expressed as mean ± standard deviation of 6 animals

*P < 0.05 **P < 0.01, significantly different from each pre-injection by Bonferroni multiple comparisons

#: In one or more animals where the ERGs were at noise level, the implicit times were unmeasurable and not suitable for statistical analysis.

PI-0.5= povidone-iodine containing 0.5% available iodine.
